# Promoter Methylation-mediated Silencing of the MiR-192-5p Promotes Endometrial Cancer Progression by Targeting ALX1

**DOI:** 10.7150/ijms.58954

**Published:** 2021-04-26

**Authors:** Jianjiao Ni, Wenjuan Tian, Shanhui Liang, Huaying Wang, Yulan Ren

**Affiliations:** 1 Department of Radiation Oncology, Fudan University Shanghai Cancer Center, Shanghai, China.; 2 Department of Gynecologic Oncology, Fudan University Shanghai Cancer Center, Shanghai, China.; 3 Department of Oncology, Shanghai Medical College, Fudan University, Shanghai, China.

**Keywords:** ALX1, endometrial carcinoma, methylation, miR-192-5p, prognosis.

## Abstract

**Background**: Epigenetic regulation by promoter methylation-mediated silencing of cancer-related microRNAs plays vital roles in tumorigenesis. MiR-192-5p promotes tumor progression in various human cancers with conflicting biological effects. However, its expression levels and biological functions in endometrial carcinoma (EC) have not been reported.

**Methods**: The methylation status of miR-192-5p in tissue samples and cell lines, was examined using bisulfite sequencing PCR. miR-192-5p expression was also measured. EC cell lines transfected with specifically designed vectors overexpressing miR-192-5p, its target gene ALX1 or both, were constructed. Tumorigenicity of these cell lines were examined by *in vitro* and *in vivo* experiments. Dual-luciferase reporter assay were employed to verify the target of miR-192-5p.

**Results**: The promoter region of miR-192-5p gene was highly methylated and its expression significantly repressed in EC samples. Moreover, a higher level of promoter methylation as well as a lower expression of miR-192-5p, was significantly associated with advanced Federation of Gynecology and Obstetrics stage and shorter disease-free survival in patients with curatively resected EC. Functional studies demonstrated that miR-192-5p overexpression inhibited *in vitro* tumor progression, *in vivo* tumorigenicity and the expression of several oncoproteins that was highly related to epithelial-to-mesenchymal transition. ALX1 was verified as a direct target of miR-192-5p and demonstrated to mediate the tumor-suppressive function of miR-192-5p.

**Conclusion**: miR-192-5p is a tumor suppressor miRNA that is epigenetically silenced by promoter methylation and may serve as a potential prognostic biomarker in EC.

## Introduction

Although early stage endometrial carcinoma (EC) has a promising survival rate, the prognosis of advanced EC is still dismal 1, 2. Two major clinic-pathological subtypes of EC exist, with the type I EC closely associated with estrogen and the type II EC mainly consisting of uterine serous carcinoma and clear cell carcinoma 3. Genetic mutations in PTEN, CTNNB1, PIK3CA, ARID1A, KRAS and ARID5B were frequently observed in endometrioid EC, while TP53 mutations as well as extensive copy number alterations were commonly detected in uterine serous carcinoma, using integrated genomic, transcriptomic and proteomic analyses 4. Additionally, PTEN/PI3K/AKT pathway, microsatellite instability, and epithelial-to-mesenchymal transition were repeatedly found to be connected with tumorigenesis of EC 5, 6. Nevertheless, the exact molecular mechanisms tumorigenesis and cancer progression of EC are largely unknown.

miRNAs, a class of small noncoding RNAs (19-25 nucleotides), frequently participate in the regulation of the expression of protein-coding genes at the post-transcription level 7, 8. Dysregulation of miRNAs are associated with carcinogenesis of various malignancies 9, including EC 10, 11, 12, 13, 14. Recently, a plenty of miRNAs, such as miR-145, miR-193a-5p, miR-204-5p, microRNA-505 and miR-200c, are identified to play important roles in tumor initiation and disease progression of EC 10, 11, 12, 13, 14. miR-192-5p (also known as miR-215-5p) , a newly characterized microRNA, is differently expressed in various human cancers and could participate in different kinds of biological processes during tumorigenesis 15,16,17. Nevertheless, the biological functions of miR-192-5p in EC have not been reported.

Epigenetic modulation through promoter methylation plays a vital role in regulating the expression and function of cancer-related miRNAs. Various miRNAs including miR-132, miR-148a, miR-107 and miR-34 are shown to be silenced by aberrant hypermethylation of CpG islands in/near the promoter region 18, 19, 20. A previous study found that miR-192-5p could be downregulated by aberrant methylation in the promoter region, which ultimately promotes disease progression in pancreatic cancer 21. In our recent study, the expression levels of 1347 common human microRNAs in two EC cell lines were detected using a specific microarray, before and after treating with demethylation agents for 72 h 22. During that study, miR-192-5p expression increased significantly after 5‑aza‑2'‑deoxycitidine treatment, with a fold-change of about 1.8 (unpublished data). Therefore, we set out to elucidate the methylation status and biological function of miR-192-5p in EC.

In the current study, we measured the methylation status of the promoter region of miR-192-5p gene and investigated its relationship with the expression level of miR-192-5p in patient's samples as well as cell lines of EC. The prognostic significance of miR-192-5p expression and its methylation status were examined in EC patients who underwent completely surgical resection. Afterwards, the biological functions and molecular targets of miR-192-5p, were investigated in depth.

## Materials and Methods

### Clinical samples

We collected 56 paired samples with both cancerous tissues and non-cancerous tissues immediately after surgical removal from EC patients who underwent completely primary resection without neoadjuvant therapy between January 2014 and April 2014 at Fudan University Shanghai Cancer Center (FUSCC). The ethical review committees at FUSCC approved this study and we obtained written informed consents from all of the patients, baseline information of whom were summarized in Supplementary Table 1.

### Cell culture, vector construction and cell transfection

We purchased two of the commonly used EC cell lines, KLE and Ishikawa (ISH), from the American type culture collection, which had already been authenticated. Vectors containing miRNA mimicking miR-192-5p, siRNA interfering ALX1, were synthesized by Shanghai GenePharma co., Ltd. Moreover, a specific vector containing the entire coding sequence of ALX1 but lacked its 3'-UTR was designed. Lentiviral systems used to transfect the various vectors were purchased and transfection was done with a final concentration of 50nM, using Lipofectamine™ 2000 Transfection Reagent (Invitrogen; Thermo Fisher Scientific, Inc.). After culturing with 1µg/ml puromycin for 14 days, successfully transduced EC cell lines were selected.

### DNA methylation analysis and demethylation treatment

Genomic DNA from tissue samples and cell lines were extracted using the QIAamp DNA formalin-fixed paraffin-embedded Tissue Kit(Qiagen, Inc.). Next, we performed sodium bisulfite modification of the DNA using the EpiTect Bisulfite Kit (Qiagen, Inc.). The promoter region of miR-192-5p was then amplified using specific primers, the sequences of which were in supplementary Table 2. Finally, the methylation status was measured by sequencing performed at GeneTech (Shanghai) co., Ltd. We treated the EC cell lines with 3μM 5-aza-2-deoxycytidine for 72 hours, as an approach of demethylation.

### RNA isolation and semi-quantitative PCR

We extracted total RNA using the TRIzol reagent (Thermo Fisher Scientific, Inc.). Small RNAs were purified using the mirVana miRNA isolation kit (Thermo Fisher Scientific, Inc.). The relative expression level of miR-192-5p was measured using the TaqMan microRNA assays (Applied Biosystems; Thermo Fisher Scientific, Inc.). Semi-quantitative PCR with SYBR green I (Takara Biotechnology co., Ltd.) was used to compare the relative expression of ALX1 mRNAs using the SYBR® Premix Ex TaqTM Kit (Takara Biotechnology co., Ltd.).

### Cell proliferation, migration and invasion assay, apoptosis analysis

Cell proliferations were investigated using CCK-8 assay and colony-formation assay. Transwell cell migration assay kits were used to examine the cell migration capacity. BioCoat™ Matrigel® Invasion Chambers (corning Incorporated) was employed to investigate the cell invasion capcacity. Cell apoptosis was analyzed with the Annexin V‑fluorescein isothiocyanate/propidium iodide Apoptosis detection kit. In brief, 1x105 cells were re-suspended in 500 µl binding buffer and stained with 5 µl Annexin V and 5 µl PI in the dark at room temperature for 20 min. The samples were then analyzed using a FACScan flow cytometer and the CellQuest™ Pro 1.0 software (BD Biosciences), according to the manufacturer's instructions.

### Western Blot analysis

Cells were harvested and lysed in cell lysis buffer and protein concentrations of the lysed extracts were measured and equalized with the extraction reagent. After loading with equal amounts of the extracts, subjecting to SDS-PDGE, transferring onto nitrocellulose membranes, protein expression were then analyzed^19^. ALX1 (ab181101, Abcam), MMP2 (40994, Cell Signaling), MMP9 (13667, Cell Signaling), VEGF (ab69479, Abcam), E-cadherin (14472, Cell Signaling), Vimentin (5741, Cell Signaling), tubulin (5568, Cell Signaling), were used as primary antibody. The secondary antibodies used included: Horseradish peroxidase-conju-gated secondary antibodies against rabbit (cat. no. SA00001-2; 1:10,000) and mouse (cat. no. SA00001-1; 1:10,000), all of which were purchased from Proteintech Group, Inc.

### *In vivo* tumorigenicity

Fifty female nude mice (4-6 weeks old) were purchased and placed under specific pathogen-free conditions, using laminar flow cabinets. After one week of adapting to the environment, a total of 1X106 cells of each experimental group were injected intraperitoneally. In detail, we randomly separated the mice into a total of seven groups in three independent experiments. In each experiment, the mice were further randomly divided into two or three groups, with each group consisting of 6 mice.

Before the procedures, the mice were anesthetized and the size of the tumor were recorded weekly. During the study, each group was consisted of six randomly selected mice. Three weeks after tumor cell implantation, the mice were euthanatized and tumor weight was measured. The Committee on the Use of Live Animals in Teaching and Research of Fudan University, approved the protocols of animal study. All the animal studies were in accordance with the ARRIVE (Animals in Research: Reporting *In Vivo* Experiments) guidelines.

### Luciferase assay

Luciferase constructs were generated by ligating oligonucleotides containing the wild-type or mutant putative target site of the ALX1 3'-UTR into the Psi-CHECK2 vector (Promega) downstream of a luciferase gene. The wild type/mutant type vectors were co-transfected with miR-192-5p mimics/negative control into 293T cell, using Lipofectamine. After 48 hours, we harvested the cell lines and detected the luciferase activity of each group, using the dual-luciferase reporter assay system, with the renilla luciferase chosen as the internal control.

### Statistical analysis

GraphPad Prism Software 7.0 and SPSS 22.0 were used for the statistical analyses. For comparisons, one-way ANOVA, t test, Pearson chi-square test were performed. Tukey's post hoc test was used for multiple comparisons test. Disease-free survival (DFS) was defined as the time interval from surgery to clinically or radiologically proven recurrence or death due to any cause. DFS was calculated by the Kaplan-Meier method, and the differences between the survival curves were examined by using the log-rank test. The correlation between miR-192-5p expression, and promoter methylation, as well as ALX1 mRNA expression, was determined by calculating the Spearman correlation coefficient, with r and p values indicated. Data was presented as the mean ± SD and considered significant when p<0.05.

## Results

### The miR-192-5p gene is methylated and downregulated in endometrial carcinoma

**Figure 1:**
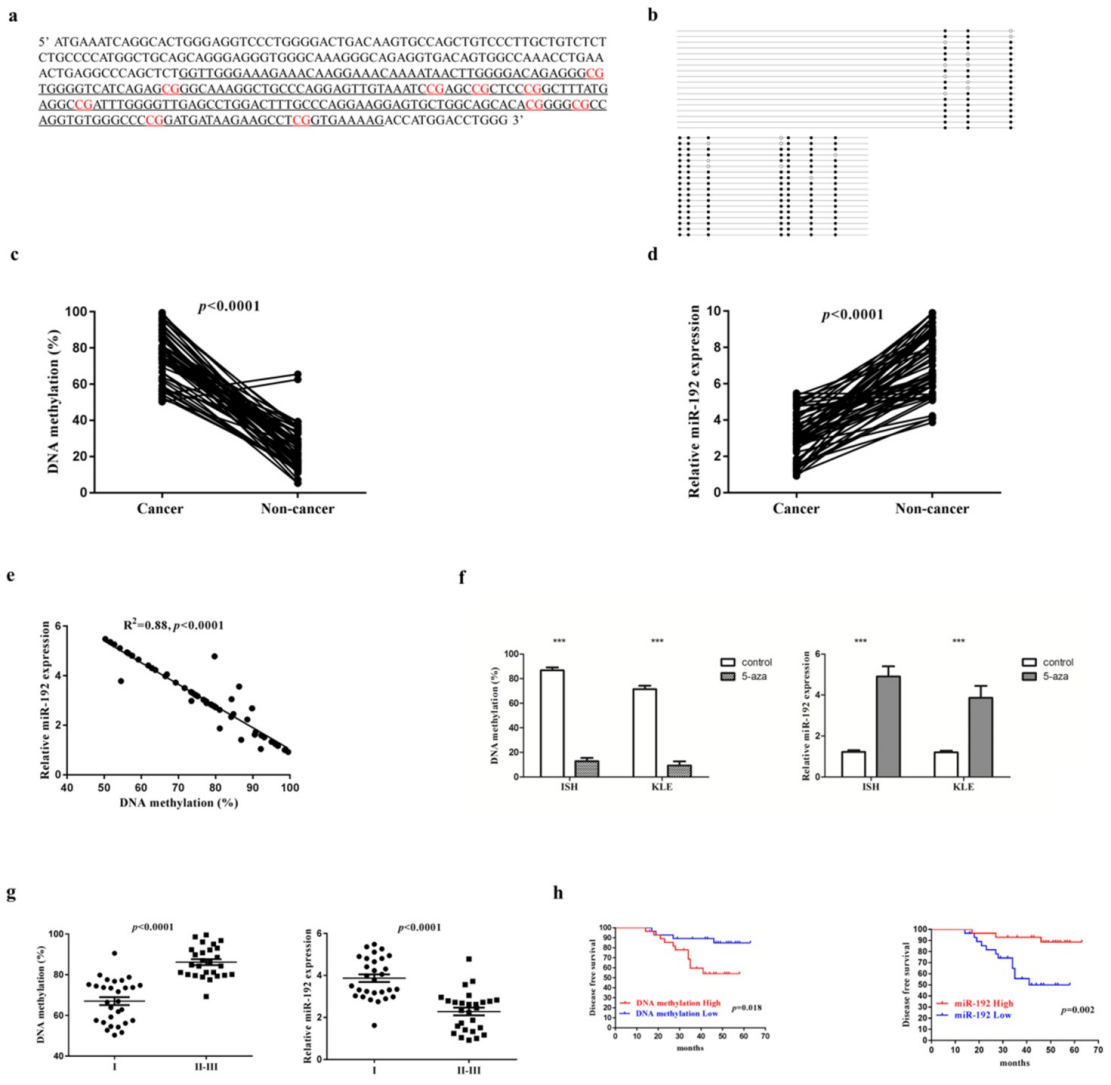
miR-192-5p is methylated and downregulated in endometrial carcinoma. (a) DNA sequence located 3.5 kb upstream of the miR-192-5p. The sequence underlined delineates the promoter region. CG dinucleotides exhibits differential methylation are in red. (b) Bisulfite sequencing analysis to determine the DNA methylation status of the promoter of miR-192-5p gene, showing the result of one patient's cancerous tissue. The DNA methylation status of 10 CpG sites were measured for 15 times and the mean value was calculated. Black dots represented methylated CpG sites, white dots represented un-methylated CpG sites. The percentage of black dots (138/150) in Figure 1b represented the relative level (88.0%) of methylation in this specific sample. The DNA methylation level of miR-192-5p (c) and relative expression of miR-192-5p (d) in 56 paired endometrial carcinoma tissues and adjacent non-cancer tissues, were assessed. The statistical significance of differences between cancer tissues and adjacent non-cancer tissues was calculated using Student's t-test. (e) The correlation between relative miR-192-5p expression and the DNA methylation level was evaluated using Spearman's correlation analysis. (f) Endometrial carcinoma cell lines were treated with 3 mM 5-aza-2'-deoxycitidine for 72 hours. And then, relative miR-192-5p expression and the DNA methylation level were examined. The results are presented as the means ± s.d. of values obtained in three independent experiments. (g) Correlations between relative miR-192-5p expression and the DNA methylation level, with FIGO stage. The statistical significance of differences between different groups was calculated using Student's t-test. (h) Disease free survival estimated by Kaplan‑Meier method. The median expression level of miR-192-5p was selected as the cut-off point. *P<0.05, **P<0.01 and ***P<0.001. miR, microRNA; EC, endometrial carcinoma; 5‑aza, 5‑aza‑2'‑deoxycitidine.

We identified one CpG-rich region at the promoter of miR-192-5p gene using CpG Island Searcher 23, which locates at chromosome 11q13.1 (Figure 1a). After designing specific primer sets, the methylation level of miR-192-5p was evaluated with bisulfite sequencing PCR (Figure 1b). The methylation status of successive CpG sites across several hundred bases was highly concordant. And thus, we used the mean value as a measure of the relative methylation levels of miR-192-5p. The frequency of miR-192-5p promoter hypermethylation in our study was 71.4% choosing 70% as the cut-off since the highest relative methylation level in the non-cancerous tissues was near 70%. When compared with that in the non-cancerous tissue, miR-192-5p gene was significantly hyper-methylated in the cancerous tissues in the 56 paired EC patient's samples (Figure 1c). Of note, representative images of paired cancerous and non-cancerous tissues from both endometrioid and serous carcinoma in our study were shown in Supplementary Figure 1.

Next, to examine the effect of DNA methylation of the miR-192-5p promoter regions on miR-192-5p expression, we measured the relative miR-192-5p expression in 56 paired EC samples by semi-quantitative PCR. We detected a significant downregulation of miR-192-5p expression in cancerous tissues, with a fold change of 2.29 (3.11±1.23 vs 7.13±1.72, p<0.001) (Figure 1d). In addition, a significant inverse correlation between miR-192-5p DNA methylation and miR-192-5p expression was demonstrated (Figure 1e). Moreover, we treated two EC cell lines with 5-aza-2'-deoxycitidine. Both cell lines displayed hypermethylation of the miR-192-5p promoter region before demethylating agent treatment, and DNA methylation levels decreased significantly after 72 hours of 5-aza-2'-deoxycitidine treatment, with the fold change of 6.7 (86.8±2.37 vs 12.9±1.65, p<0.0001) In ISH and 7.6 (71.4±2.19 vs 9.37±1.32, p<0.0001) in KLE. Moreover, the miR-192-5p expression increased significantly after 72 hours of 5-aza-2'-deoxycitidine treatment, with the fold change of 4.0 (1.22±0.08 vs 4.91±0.49, p<0.001) In ISH and 3.2 (1.20±0.06 vs 3.86±0.27, p<0.001) in KLE (Figure 1f).

Finally, correlations between miR-192-5p methylation as well as miR-192-5p expression levels, with age, primary tumor size, Federation of Gynecology and Obstetrics (FIGO) stage, tumor differentiation and tumor histology, were examined. A significant positive correlation between miR-192-5p promoter methylation level and FIGO stage was found, with a mean level of relative promoter methylation of 67.0 ±10.34 and 86.2 ±72.8 (p<0.0001) among patients with FIGO stage I disease and FIGO II-III disease, respectively (Figure 1g). Additionally, a significant negative correlation between relative miR-192-5p expression with FIGO stage was also demonstrated, with a mean level of relative miR-192-5p expression of 3.87 ±0.95 and 2.28 ±0.93 (p<0.0001) among patients with FIGO stage I disease and FIGO II-III disease, respectively (Figure 1g). No other significant relationship was found. Moreover, a higher level of promoter methylation (p=0.018), as well as a lower expression of miR-192-5p (p=0.002), were significantly associated with shorter disease-free survival (n=56), when the median values were chosen as the cut-off (Figure 1h).

### miR-192-5p functions as a tumor suppressive microRNA in endometrial carcinoma

**Figure 2:**
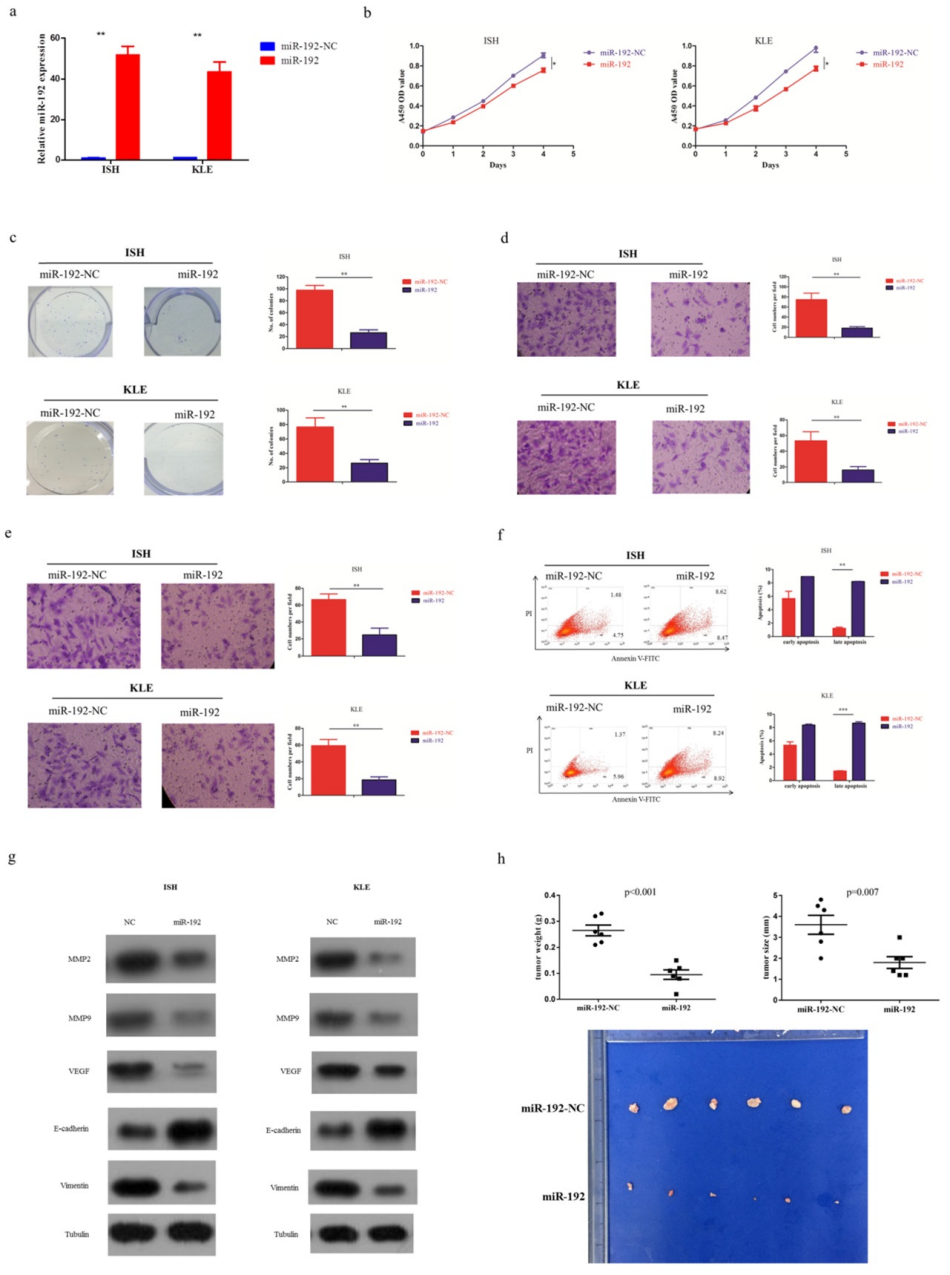
miR-192-5p function as a tumor suppressive microRNA in endometrial carcinoma. Two endometrial carcinoma cell lines, ISH and KLE, were successfully transduced with miR-192-5p lentiviral vector (miR-192) or its negative control (miR-192-NC), respectively. (a) Relative miR-192-5p expression were examined. (b, c ) Cell proliferation was examined by CCK-8 assay (b) and colony formation (c). (d, e) Cell migration and invasion were investigated by migration assays (d) and invasion assays (e), respectively. The number of cells that invaded through the membrane was counted in 10 fields under the x20 objective lens. (f) At 48 hours after transfection, apoptosis was measured by flow cytometric analysis of cells stained with Annexin V-FITC and PI. (g) Common oncoproteins were subjected to western blotting using the indicated antibodies. (h) *In vivo* tumorigenicity investigated in in a nude mouse xenograft model. Tumor size and weight were measured 3 weeks after cancer cell injection. All of the results are presented as the means ± s.d. of values obtained in three independent experiments. Statistical significance was calculated using the Student's t-test. *p<0.05, **p<0.01,***p<0.001.

EC cell lines transfected with miR-192-5p mimics (miR-192) or its corresponding negative control (miR-192-NC), were constructed (Figure 2a). Overexpression of miR-192-5p inhibited cell proliferation in EC cell lines, using CCK-8 assay (Figure 2b) and colony formation assay (Figure 2c). It also suppressed cell migration (Figure 2d) and invasion (Figure 2e). Additionally, miR-192-5p overexpression promoted cell apoptosis in EC cell lines and we observed >6-fold increases in late apoptotic cell numbers in the “miR-192 group” when compared with that in its corresponding negative control group (Figure 2f). Moreover, miR-192-5p overexpression was found to result in the downregulation of MMP2, MMP9, VEGF and Vimentin, but upregulation of E-cadherin (Figure 2g).

To further determine the tumor suppressive effect of miR-192-5p *in vivo*, EC cell lines overexpressing miR-192-5p as well as its negative control, were intraperitoneally injected into nude mice. In the baseline, each group had 6 mice with good health. As expected, miR-192-5p overexpression significantly reduced tumor growth in nude mice (Figure 2h).

### miR-192-5p directly targets ALX1

**Figure 3:**
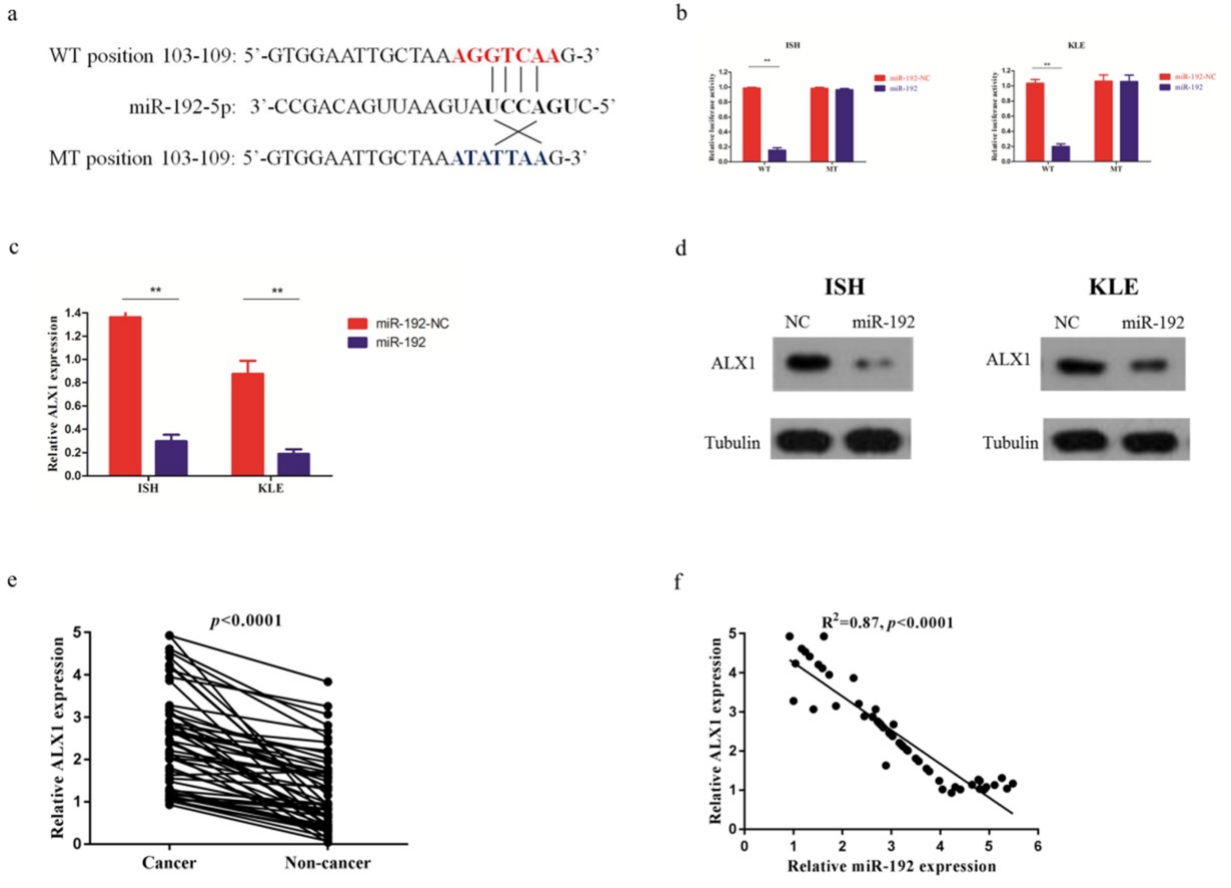
miR-192-5p downregulates ALX1 expression by directly targeting its 3'-UTR. (a) Human ALX1 3'UTR fragment containing the wild-type or mutant miR-192-5p-binding sequence. (b) 3'-UTR luciferase reporter assays in 293 T cells. 293 T cells were co-transfected with miR-192-5p or its negative control vector and a luciferase reporter construct containing the wild-type or mutant ALX1 3'UTR. For each experiment, the data were normalized to the luciferase activity detected in cells transfected with the control vector. (c, d) Relative ALX1 mRNA expression and protein expression in ISH and KLE cell lines that were stably transfected with miR-192-5p lentiviral vector (miR-192) or its negative control (miR-192-NC). (e) Relative expression of ALX1 mRNA in 56 paired endometrial carcinoma tissues and adjacent non-cancer tissues were assessed by real-time PCR. GAPDH served as an internal control. (f) The correlation between relative miR-192-5p expression and relative ALX1 mRNA expression was evaluated using Spearman's correlation analysis.

After searching in the commonly used microRNA databases, including DIANA-microT-CDS, TargetScan, and microrna, ALX1 was found to be one of the targets. To validate this prediction, the entire wild-type 3'-UTR of ALX1 or the mutant 3'-UTR with a 4-bp mutation in the seed region was cloned downstream of the luciferase gene's open reading frame (Figure 3a). The luciferase activities significantly decreased upon transfection of miR-192-5p mimics in the wild type reporter, with a fold change of 6.2 (0.99±0.03 vs 0.16±0.01, p<0.001). Conversely, the luciferase activities of mutant reporter was unaffected upon transfection with either miR-192-5p mimics or the control vector, with the relative luciferase activity of 0.98±0.01 and 0.96±0.02 (p=0.167) (Figure 3b). In addition, the ALX1 mRNA level (Figure 3c) and protein level (Figure 3d), were substantially reduced after miR-192-5p overexpression in both EC cell lines. The fold changes of ALX1 mRNA after miR-192 overexpression were 4.5 (1.36±0.12 vs 0.30 ±0.06, p=0.01) and 4.6 (0.88±0.11 vs 0.19±0.04, p=0.01) in ISH and KLE, respectively. Furthermore, in the 56 paired EC samples, ALX1 expression were significantly elevated in the cancerous tissues, compared with that in the non-cancerous tissues (Figure 3e) and we found a significant inverse correlation between relative ALX1 mRNA level and relative miR-192-5p expression (Figure 3f).

### miR-192-5p mediates tumor suppressive effect through ALX1 in endometrial carcinoma

**Figure 4:**
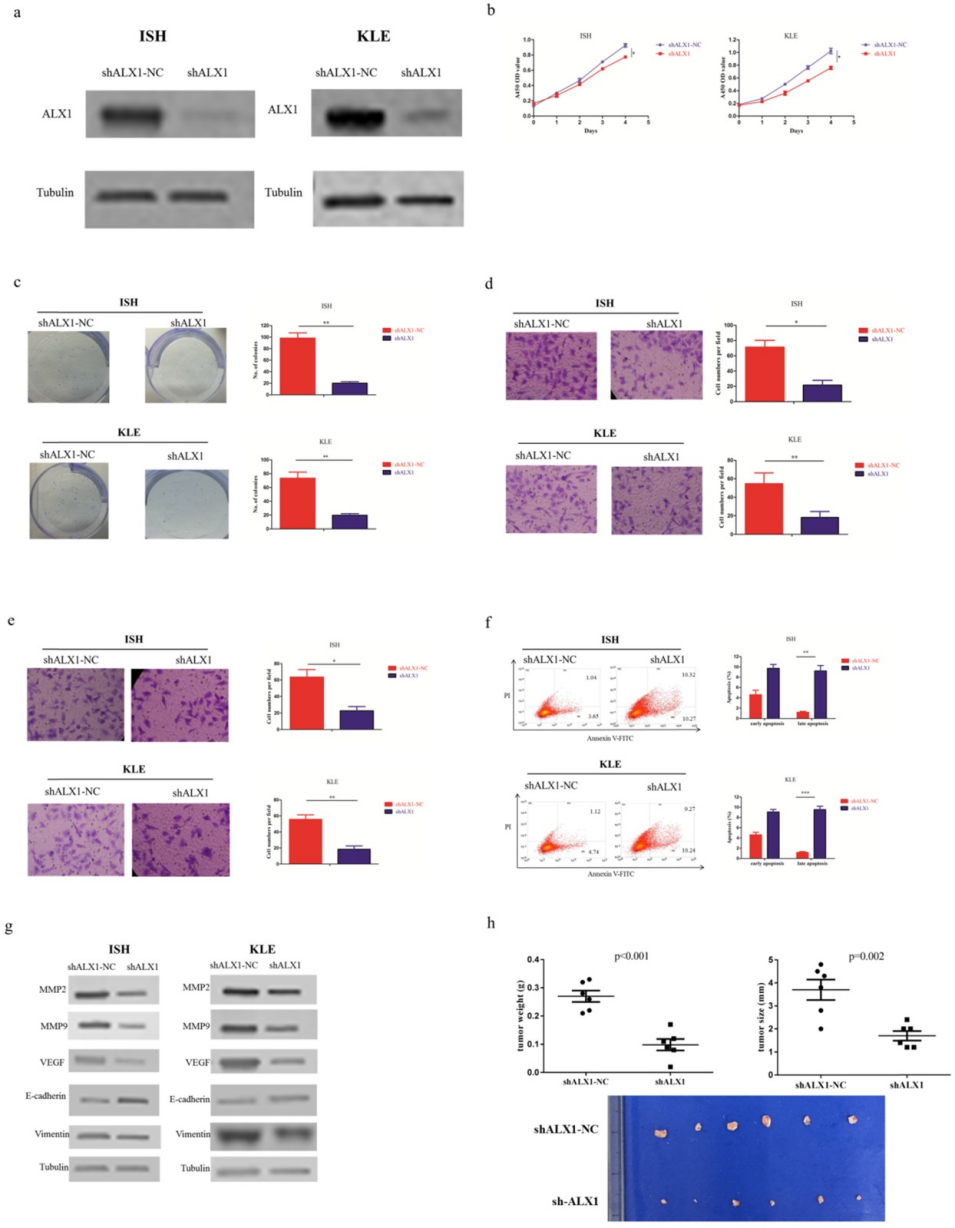
ALX1 functions as a onco-protein in endometrial carcinoma. Two endometrial carcinoma cell lines, ISH and KLE, were stably transfected with shALX1 lentiviral vector (shALX1) or its negative control (shALX1-NC), respectively. (a) ALX1 expression were examined by western blotting using the indicated antibodies. (b, c ) Cell proliferation was examined by CCK-8 assay (b) and colony formation (c). (d, e) Cell migration and invasion were investigated by migration assays (d) and invasion assays (e), respectively. The number of cells that invaded through the membrane was counted in 10 fields under the x20 objective lens. (f) At 48 hours after transfection, apoptosis was measured by flow cytometric analysis of cells stained with Annexin V-FITC and PI. (g) MMP2, MMP9, VEGF, E-cadherin and Vimentin expression, were subjected to western blotting using the indicated antibodies. Tubulin expression was chosen as a control. (h) *In vivo* tumorigenicity investigated in in a nude mouse xenograft model. Tumor size and weight were measured 3 weeks after cancer cell injection. All of the results are presented as the means ± s.d. of values obtained in three independent experiments. Statistical significance was calculated using the Student's t-test. *p<0.05, **p<0.01,***p<0.001.

In order to investigate whether ALX1 was essential for the antitumor effects of miR-192-5p, we infected ISH and KLE cell lines with lentivirus encoding shALX1, which mediated stable knockdown of endogenous ALX1 (Figure 4a). The biological effects of ALX1 downregulation mimicked that of miR-192-5p overexpression (Figure 4b-4h).

**Figure 5:**
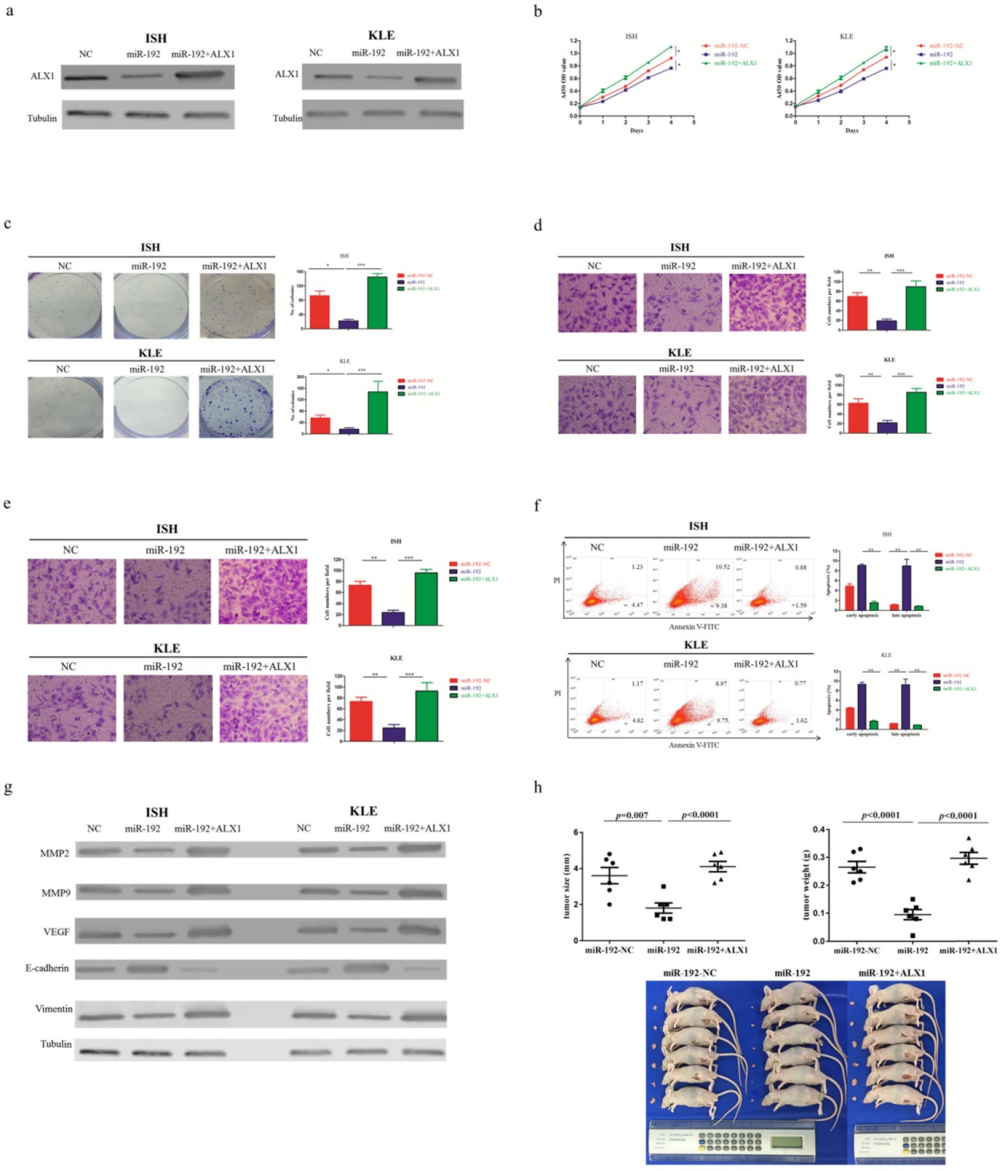
ALX1 mediates the tumor-suppressive function of miR-192-5p. Three special cell lines were used in these experiments: EC cell lines transfected with negative control (miR-192-NC), miR-192-5p mimics (miR-192), and both miR-192-5p mimics and a ALX1-expressing vector that encoded the entire coding sequence of ALX1 but lacked the 3'-UTR (miR-192+ALX1). (a) ALX1 expression were examined by western blotting using the indicated antibodies. (b, c ) Cell proliferation was examined by CCK-8 assay (b) and colony formation (c). (d, e) Cell migration and invasion were investigated by migration assays (d) and invasion assays (e), respectively. The number of cells that invaded through the membrane was counted in 10 fields under the x20 objective lens. (f) At 48 hours after transfection, apoptosis was measured by flow cytometric analysis of cells stained with Annexin V-FITC and PI. (g) MMP2, MMP9, VEGF, E-cadherin and Vimentin expression, were subjected to western blotting using the indicated antibodies. Tubulin expression was chosen as a control. (h) *In vivo* tumorigenicity investigated in in a nude mouse xenograft model. Tumor size and weight were measured 3 weeks after cancer cell injection. All of the results are presented as the means ± s.d. of values obtained in three independent experiments. Statistical significance was calculated using the Tukey's post hoc test. *p<0.05, **p<0.01,***p<0.001.

Next, a rescue experiment was carried out to further examine the biological and molecular relations between ALX1 and miR-192-5p in EC. We co-transfected EC cell lines with miR-192-5p mimics and a specific lentiviral vector that contained the entire coding sequence of ALX1 but lacked its 3'-UTR (miR-192+ALX1), in order to ectopically express ALX1 without interference of miR-192-5p and its many other targets. Using these three specially designed EC cell lines (ie: miR-192-NC, miR-192, miR-192+ALX1), we demonstrated that ectopic expression of ALX1 significantly abrogated the tumor-suppressive effect induced by miR-192-5p (Figure 5). Taken together, these data indicate that miR-192-5p mediates tumor suppressive effect through ALX1 in EC.

## Discussion

Accumulating evidence suggest that miR-192-5p plays vital roles in tumorigenesis of various human cancers 24, 25, 26 but its exact molecular functions remain conflicting in different cases. In this study, we demonstrated that miR-192-5p is downregulated through promoter hypermethylation and functions as a powerful tumor suppressive microRNA by targeting ALX1 in endometrial carcinoma.

The exact biological role of miR-192-5p in human cancers is still controversial. Previous studies identified miR-192-5p as a tumor suppressive microRNA in renal cancers 27, 28, lung cancer 29, ovarian carcinoma 26 and hepatocellular carcinoma 24, 26. However, miR-192-5p was found to promote tumor progression in gastric cancer 25, 30, prostate cancer 31, ovarian carcinomas 17 and lung cancer 32. In our study, miR-192-5p was shown to be downregulated by promoter hypermethylation and demonstrated to be a tumor suppressive microRNA in EC. Normally, one microRNA can regulate the expression of various protein-coding genes and can be evolved in a plenty of complicated signaling networks. In different cancer types, the driver mutations and aberrant proteins are distinct, which may dictate diverse signaling pathways evolving the same microRNA, making it possible that the same microRNA plays opposite roles in different cancers.

ALX1 is a homeobox transcription factor recently recognized as a novel regulator of epithelial-to-mesenchymal transition. Expression and activation of ALX1, were shown to induce the expression of snail in ovarian cancers 33, and promote tumor progression in osteosarcoma 34. ALX1 was also frequently found to have important prognostic significances in quite a few human cancers, including melanoma, gastric cancer, lung cancer, kidney cancer, breast cancer and liver carcinoma 35, 36, 37. In our study, ALX1 overexpression promoted tumor progression in EC at least partly through upregulation of MMP2, MMP9, VEGF and vimentin. All of these biological functions led to the hypothesis that ALX1 possibly participated in the regulation of epithelial-to-mesenchymal transition in EC, which warranted further investigation.

MicroRNA mediated repression of the expression of protein-coding genes at the post-transcription level, is a common epigenetic mechanism occurring in various kinds of physiological and pathological processes 8, 9. As a newly characterized homeobox transcription factor, few microRNA regulating ALX1 has been identified. Stepicheva NA et al found that miR-31 blocked the expression of ALX1 in primary mesenchyme cells, which was critical for skeletal patterning in the sea urchin embryo 38. However, using the microRNA databases, such as DIANA-microT-CDS, TargetScan, and microrna, a large amount of microRNAs (including miR-192-5p) could be found that were predicted to have the potential to directly targeting ALX1. In the current study, ALX1 was demonstrated to be one of the direct targets of miR-192-5p through luciferase assay, expression correlation and biological rescue experiments. On the other hand, a plenty of targets of miR-192-5p, such as X-linked inhibitor of apoptosis (XIAP), PABPC4 and ERCC3/4, have been discovered previously 29, 39, 40.

Recent work has begun to shed light on the importance of epigenetic modifications of microRNAs. Various tumor suppressing microRNAs have been shown to be silenced by aberrant methylation of their promoter CpG islands 18, 19, 20, 41, 42, 43. In the present study, the promoter region of miR-192-5p gene was shown to be hyper-methylated in EC, which is consistent with previous studies focusing miR-192-5p in hepatocellular carcinoma 24 and pancreatic cancer 21. Moreover, the methylation status of the promoter of miR-192-5p, as well as the relative expression of miR-192-5p, was found to be associated with FIGO stage and disease-free survival in EC patients, highlighting the crucial prognostic significance of miR-192-5p in EC.

## Conclusions

miR-192-5p is silenced by promoter hypermethylation, which lead to the overexpression of ALX1 and promotion of tumor progression in EC.

## Supplementary Material

Supplementary tables.

## References

[1] Bray F, Ferlay J, Soerjomataram I (2018). Global cancer statistics 2018: GLOBOCAN estimates of incidence and mortality worldwide for 36 cancers in 185 countries. CA Cancer J Clin.

[2] Philippe M, Alexandra L, Carien C (2016). Endometrial cancer. Lancet.

[3] Jonathan LH, George LM (2006). Molecular and pathologic aspects of endometrial carcinogenesis. J Clin Oncol.

[4] Cancer Genome Atlas Research Network, Kandoth C, Schultz N (2013). Integrated genomic characterization of endometrial carcinoma. Nature.

[5] Silva JL, Paulino E, Dias MF (2015). Endometrial cancer: redefining the molecular-targeted approach. Cancer Chemother Pharmacol.

[6] Bell DW, Ellenson LH (2019). Molecular Genetics of Endometrial Carcinoma. Annu Rev Pathol.

[7] Jonas S, Izaurralde E (2015). Towards a molecular understanding of microRNA-mediated gene silencing. Nat Rev Genet.

[8] Bartel DP (2009). MicroRNAs: target recognition and regulatory functions. Cell.

[9] Bracken CP, Scott HS, Goodall GJ (2016). A network-biology perspective of microRNA function and dysfunction in cancer. Nat Rev Genet.

[10] Wu Y, Liu S, Xin H (2011). Up-regulation of microRNA-145 promotes differentiation by repressing OCT4 in human endometrial adenocarcinoma cells. Cancer.

[11] Yang Y, Zhou L, Lu L (2013). A novel miR-193a-5p-YY1-APC regulatory axis in human endometrioid endometrial adenocarcinoma. Oncogene.

[12] Bao W, Wang HH, Tian FJ (2013). A TrkB-STAT3-miR-204-5p regulatory circuitry controls proliferation and invasion of endometrial carcinoma cells. Mol Cancer.

[13] Chen S, Sun KX, Liu BL (2016). MicroRNA-505 functions as a tumor suppressor in endometrial cancer by targeting TGF-alpha. Mol Cancer.

[14] Li Q, Zhang C, Chen R (2016). Disrupting MALAT1/miR-200c sponge decreases invasion and migration in endometrioid endometrial carcinoma. Cancer Lett.

[15] Ge Y, Yan X, Jin Y (2015). MiRNA-192 [corrected] and miRNA-204 Directly Suppress lncRNA HOTTIP and Interrupt GLS1-Mediated Glutaminolysis in Hepatocellular Carcinoma. PLoS Genet.

[16] Chen Z, Han S, Huang W (2016). MicroRNA-215 suppresses cell proliferation, migration and invasion of colon cancer by repressing Yin-Yang 1. Biochem Biophys Res Commun.

[17] Agostini A, Brunetti M, Davidson B (2018). The microRNA miR-192/215 family is upregulated in mucinous ovarian carcinomas. Sci Rep.

[18] Lujambio A, Ropero S, Ballestar E (2007). Genetic unmasking of an epigenetically silenced microRNA in human cancer cells. Cancer Res.

[19] Wang P, Chen L, Zhang J (2014). Methylation-mediated silencing of the miR-124 genes facilitates pancreatic cancer progression and metastasis by targeting Rac1. Oncogene.

[20] Loginov VI, Rykov SV, Fridman MV (2015). Methylation of miRNA genes and oncogenesis. Biochemistry (Mosc).

[21] Botla SK, Savant S, Jandaghi P (2016). Early Epigenetic Downregulation of microRNA-192 Expression Promotes Pancreatic Cancer Progression. Cancer Res.

[22] Ni J, Liang S, Shan B (2020). Methylationassociated silencing of miR638 promotes endometrial carcinoma progression by targeting MEF2C. Int J Mol Med.

[23] Takai D, Jones PA (2003). The CpG island searcher: a new WWW resource. In Silico Biol.

[24] Gu Y, Wei X, Sun Y (2019). miR-192-5p silencing by genetic aberrations is a key event in hepatocellular carcinomas with cancer stem cell features. Cancer Res.

[25] Zhang X, Peng Y, Huang Y (2018). Inhibition of the miR-192/215-Rab11-FIP2 axis suppresses human gastric cancer progression. Cell Death Dis.

[26] Wu SY, Rupaimoole R, Shen F (2016). A miR-192-EGR1-HOXB9 regulatory network controls the angiogenic switch in cancer. Nat Commun.

[27] Senanayake U, Das S, Vesely P (2012). miR-192, miR-194, miR-215, miR-200c and miR-141 are downregulated and their common target ACVR2B is strongly expressed in renal childhood neoplasms. Carcinogenesis.

[28] Khella HW, Bakhet M, Allo G (2013). miR-192, miR-194 and miR-215: a convergent microRNA network suppressing tumor progression in renal cell carcinoma. Carcinogenesis.

[29] Ye M, Zhang J, Zhang J (2015). Curcumin promotes apoptosis by activating the p53-miR-192-5p/215-XIAP pathway in non-small cell lung cancer. Cancer Lett.

[30] Zhang X, Peng Y, Huang Y (2018). SMG-1 inhibition by miR-192/-215 causes epithelial-mesenchymal transition in gastric carcinogenesis via activation of Wnt signaling. Cancer Med.

[31] Chen ZJ, Yan YJ, Shen H (2019). miR-192 is Overexpressed and Promotes Cell Proliferation in Prostate Cancer. Med Princ Pract.

[32] Filipska M, Skrzypski M, Czetyrbok K (2018). MiR-192 and miR-662 enhance chemoresistance and invasiveness of squamous cell lung carcinoma. Lung Cancer.

[33] Yuan H, Kajiyama H, Ito S (2013). ALX1 induces snail expression to promote epithelial-to-mesenchymal transition and invasion of ovarian cancer cells. Cancer Res.

[34] Yang M, Pan Y, Zhou Y (2015). Depletion of ALX1 causes inhibition of migration and induction of apoptosis in human osteosarcoma. Tumour Biol.

[35] Ohara K, Arai E, Takahashi Y (2017). Genes involved in development and differentiation are commonly methylated in cancers derived from multiple organs: a single-institutional methylome analysis using 1007 tissue specimens. Carcinogenesis.

[36] Rambow F, Job B, Petit V (2015). New Functional Signatures for Understanding Melanoma Biology from Tumor Cell Lineage-Specific Analysis. Cell Rep.

[37] Sandoval J, Mendez-Gonzalez J, Nadal E (2013). A prognostic DNA methylation signature for stage I non-small-cell lung cancer. J Clin Oncol.

[38] Stepicheva NA, Song JL (2015). microRNA-31 modulates skeletal patterning in the sea urchin embryo. Development.

[39] Gu Y, Wei X, Sun Y (2019). miR-192-5p Silencing by Genetic Aberrations Is a Key Event in Hepatocellular Carcinomas with Cancer Stem Cell Features. Cancer Res.

[40] Xie X, Huang N, Zhang Y (2019). MiR-192-5p reverses cisplatin resistance by targeting ERCC3 and ERCC4 in SGC7901/DDP cells. J Cancer.

[41] Johnson TG, Schelch K, Cheng YY (2018). Dysregulated Expression of the MicroRNA miR-137 and Its Target YBX1 Contribute to the Invasive Characteristics of Malignant Pleural Mesothelioma. J Thorac Oncol.

[42] Barros-Silva D, Costa-Pinheiro P, Duarte H (2018). MicroRNA-27a-5p regulation by promoter methylation and MYC signaling in prostate carcinogenesis. Cell Death Dis.

[43] Mei Q, Li X, Zhang K (2017). Genetic and Methylation-Induced Loss of miR-181a2/181b2 within chr9q33.3 Facilitates Tumor Growth of Cervical Cancer through the PIK3R3/Akt/FoxO Signaling Pathway. Clin Cancer Res.

