# Supplementary material for: Promoter Methylation-mediated Silencing of the MiR-192-5p Promotes Endometrial Cancer Progression by Targeting ALX1
Source: Int J Med Sci. 2021 Apr 26;18(12):2510–20. doi: 10.7150/ijms.58954 (PMC8176185; doi:10.7150/ijms.58954)
Supplement: Supplementary file 1 — Supplementary tables. [file ijmsv18p2510s1.pdf]

Supplementary Table 1. Disease characteristics of patients with endometrial carcinoma (n=56)

	**n**	**%**
**Age (years)**		
median	52	
range	48-73	
**Histology**		
endometrioid adenocarcinoma	50	89.3
Serous carcinoma	6	10.7
**Histopathological grade**		
G1	7	12.5
G2, 3	49	87.5
**Primary tumor size (cm)**		
<3.5	23	41.1
>3.5	33	58.9
**FIGO stage**		
I	29	51.8
II-III	27	48.2

FIGO, Federation of Gynecology and Obstetrics

Supplementary Table 2. Primers used in this study.

Name	Primer Sequence
**Primers for qRT-PCR**	
miRNA Universal R	GTGCAGGGTCCGAGGT
miR-192-F	GCGCGCTGACCTATGAATTG
miR-192-R	AGTGCAGGGTCCGAGGTATT
U6-F	CTCGCTTCGGCAGCACA
U6-R	AACGCTTCACGAATTTGCGT
ALX1-F	TAGGGAATTGGTTGGTATTAGTA
ALX1-R	CCCAAAAAACCAAATACATTAAC
GAPDH-F	GGGAGCCAAAAGGGTCATCATCTC
GAPDH-R	CCATGCCAGTGAGCTTCCCGTTC
**Primers for bisulfite sequencing PCR**	
miR-192-5p-F	ATGAAATTAGGTATTGGGAGGTT
miR-192-5p-R	CCCAAATCCATAATCTTTTCAC
**Primers for ALX1 3'UTR cloning**	
3’UTR –F	ATGAGCTCTGCATTTGAAACAAAGCCAGAGT
3’UTR –R	GCAAGCTTAGACTGGCACATGGTAAACCTATT

Abbreviations: RT, reverse transcription primer; F, forward primer; R, reverse primer; S, sequencing primer; UTR, untranslated region.
